# Multiple Myeloma Concomitant with AL Amyloidosis: Histopathological Aspects of the Common Plasma Cell Spectrum

**DOI:** 10.3390/ijms27115120

**Published:** 2026-06-05

**Authors:** Zarina Gioeva, Liudmila Mikhaleva, Aslan Tsutsaev, Anna Tebenkova, Nikita Gutyrchik, Nikolay Shakhpazyan, Alexander Ilyichev, Lev Kakturskij

**Affiliations:** Avtsyn Research Institute of Human Morphology of Federal State Budgetary Scientific Institution “Petrovsky National Research Centre of Surgery”, 117418 Moscow, Russia; mikhalevalm@yandex.ru (L.M.); me@tsutsaev.ru (A.T.); tebenkova.ann@yandex.ru (A.T.); gyt94@yandex.ru (N.G.); alex730110@gmail.com (A.I.); levkaktur@mail.ru (L.K.)

**Keywords:** multiple myeloma, plasma cell myeloma, AL amyloidosis, bone marrow, autopsy, immunohistochemistry, histopathology

## Abstract

Concurrent multiple myeloma (MM) and AL amyloidosis is associated with the poorest outcomes among plasma cell dyscrasias and has dramatically reduced median overall survival. Despite their clinical significance, comprehensive systematic histopathological studies, characterizing multiorgan involvement and lesion severity are remarkably scarce. This study includes 24 autopsies (of 18 women and six men; median age—68 years) with MM-AL. Immunohistochemical (IHC) typing was performed with an expanded antibody panel targeting the amyloid precursor protein; anti-human CD138 antibody was used to identify plasma cells in bone marrow sections. Clinical diagnosis of MM with monoclonal G-lambda secretion, Durie–Salmon Stages II–III, was established in 17 (71%) patients; MM with monoclonal G-kappa secretion, Stage III, in five (21%); and non-secreting MM in two (8%). Systemic amyloidosis was revealed during life in only 15 (62.5%) patients. In all cases, extensive amyloid deposits were observed in the myocardium, lungs and kidneys, establishing the morphological basis for multiorgan failure. IHC typing of amyloids confirmed 18 (75%) cases of AL-lambda amyloidosis and six (25%) of AL-kappa amyloidosis. Our results clarify MM-AL morphogenesis and underscore that AL is frequently underdiagnosed in MM patients. Comprehensive histopathological studies with IHC typing are necessary to confirm the diagnosis, refine the prognosis, and optimize the therapeutic strategies.

## 1. Introduction

Multiple myeloma (MM) is a B-cell malignancy characterized by the expansion of plasma cells producing monoclonal immunoglobulins. MM is generally reported to account for approximately 1% of all cancer types and roughly 10–15% of all hematologic malignancies. MM is recognized as the second most common blood cancer following non-Hodgkin lymphoma [1,2].

However, despite significant advancements in the therapeutic landscape, including novel treatment options, MM remains uncurable in most cases. The median overall survival for MM patients falls within the 5- to 10-year range, depending heavily on the malignancy stage and molecular characteristics [3,4].

According to the 2016 *World Health Organization (WHO) Classification of Tumours of Haematopoietic and Lymphoid Tissues*, the term “multiple myeloma” was replaced with the term “plasma cell myeloma,” representing a disease characterized by the multifocal proliferation of neoplastic plasma cells in the bone marrow, often accompanied by the production of monoclonal immunoglobulin (M-protein). Then, in 2022, two new, often divergent, classifications of hematologic malignancies were published: the fifth Edition of the *World Health Organization Classification of Haematolymphoid Tumours* (WHO-HAEM5) and the International Consensus Classification (ICC) of Myeloid Neoplasms and Acute Leukemias [5,6,7]. The 2022 ICC strongly supported using the term “multiple myeloma” over “plasma cell myeloma.” Experts recognized that multiple myeloma was more common and firmly established in clinical practice.

Recent studies demonstrate that 10–15% of MM patients develop overt AL amyloidosis, a severe systemic disease that produces misfolded monoclonal immunoglobulin light chains, aggregating into amyloid fibrils and depositing extracellularly in organs [8,9].

The co-occurrence of MM and AL amyloidosis represents a complex scenario within plasma cell dyscrasias. Patients with concurrent disorders typically face poor outcomes and have dramatically lower median overall survival (OS) than those with either condition alone. The reported median OS in some cohorts of MM-AL patients is as low as 10–25 months, and, in cases with cardiac involvement, it often falls within 6–8 months. The key prognostic factor that significantly worsens the outcome in patients with concomitant MM and AL amyloidosis is the progressive dysfunction of organs caused by the deposition of amyloid fibrils. These fibrils are produced by the same clonal plasma cell population responsible for the myeloma paraprotein.

Pathogenetically, primary (de novo) systemic AL amyloidosis and MM-secondary AL amyloidosis represent different stages in the plasma cell clone evolution across the spectrum of monoclonal gammopathies. In primary systemic AL amyloidosis, the plasma cell clone is typically small (<10% of the bone marrow cells), presenting a low tumor burden and a cytogenetic profile closely resembling that of monoclonal gammopathy of undetermined significance (MGUS). The key determinant in primary systemic AL amyloidosis is the amyloidogenic potential of the light chain itself, associated with germline gene selection, somatic mutation profiles, and organ tropism, rather than the intrinsic aggressiveness of the tumor clone.

In MM-secondary AL amyloidosis, the amyloidogenic clone emerges against a pre-existing multiple myeloma, characterized by high tumor burden, typical MM cytogenetic abnormalities, and pronounced CRAB criteria. In this case, organ damage is driven by two distinct mechanisms: the direct, tumor-related effects of the myeloma (such as osteolysis, cytopenias, and renal failure) and the deposition of amyloid fibrils. The pathogeneses of primary and multiple myeloma-associated AL amyloidosis differ fundamentally: in primary AL amyloidosis, the central role is played by an early, small plasma cell clone expressing highly amyloidogenic light chains. Conversely, MM-secondary AL amyloidosis is driven by a late stage of clonal evolution, involving highly proliferative and genetically complex plasma cells. Consequently, amyloidosis acts as an additional, concurrent manifestation of the underlying malignancy.

Though the co-existence of MM and AL amyloidosis is a clinically significant scenario, the investigation of this comorbidity is often limited to small clinical series and individual case reports, in which the morphology merely confirms the presence of amyloid deposits. The lack of comprehensive, systemic studies in MM-AL patients with in-depth histopathological examination highlights a crucial gap, as research frequently focuses on single-organ involvement rather than the systemic impact of these co-occurring diseases.

The goal of our study is to fill this gap by presenting a detailed description of autopsy-based histopathology in patients with co-existing MM and AL amyloidosis. In our research, a comprehensive morphological analysis, combining histochemical and immunohistochemical techniques, was used. This approach offers significant benefits, providing essential verification of amyloid deposits, mapping their distribution patterns in organs, quantifying tissue damage, and enabling assessment of the correlation between morphological lesions and clinical symptoms.

## 2. Results

The autopsy study analyzed 24 cases (18 women and six men), with a median age of 68 years (range 63–71 years of age). Females predominated, representing 75% of the cases.

Clinical diagnosis of MM with monoclonal G-lambda secretion and widespread osteolytic lesions, Durie–Salmon Stage II (*n* = 8) and Stage III (*n* = 9), was established in 17 (71%) studied cases. Five patients (21%) presented with MM characterized by monoclonal G-kappa secretion and widespread osteolytic lesions, corresponding to Durie–Salmon Stage II. Systemic amyloidosis was diagnosed during life in 15 (62.5%) of 24 patients.

### Histopathological Findings

**Heart:** Gross examination at autopsy revealed that the heart weights ranged from 500 to 750 g, with features of eccentric hypertrophy present in seven cases. The left ventricular wall was markedly hypertrophied (wall thickness up to 3.0 cm) and associated with right ventricular wall enlargement (up to 0.9 cm).

**Figure 1:**
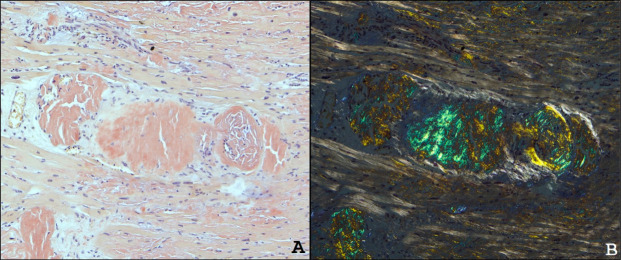
Histology of the myocardium of patient “V”, 73 years of age. (**A**) Extensive homogenous interstitial amyloid deposits demonstrating brick-red color with CR staining. (**B**) Characteristic birefringence of amyloid masses under polarized light. (**A**,**B**) ×100.

Microscopic examination of the heart demonstrated multifocal amyloid deposits accompanied by cardiomyocyte compression and atrophic foci (compression atrophy). Amyloids were also identified in vascular walls and subendocardial regions (Figure 1). Other findings included interstitial edema, multiple small-sized foci of cardiosclerosis, lipofuscin deposition, and focal areas of hyper-relaxed sarcomeres.

**Lungs:** Gross findings in the lungs included increased weight (900–1600 g) and emphysematous changes predominantly localized to the upper zones. The parenchyma exhibited a non-homogenous appearance with small focal hemorrhages and frothy dark serosanguinous fluid on the cut surface.

Microscopic examination of lung tissues demonstrated thickened interalveolar septa resulting from edema and amyloid fibril deposition within the connective tissue and vascular walls, associated with significant infiltration of lymphocytes and macrophages. Pulmonary alveoli were filled with proteinaceous masses. Lymphoid and plasma cell infiltration, organized as perivascular cords or nodular aggregates, was observed along the vascular adventitia.

**Figure 2:**
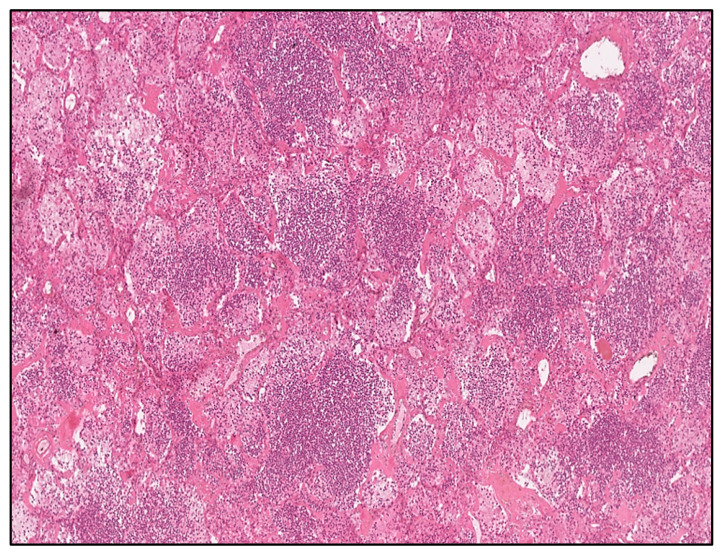
Lung tissues of patient “C”, 74 years of age. Polysegmental pneumonia. Marked infiltration of lymphocytes, neutrophils, and macrophages in the alveolar spaces and interalveolar septa. H&E staining, ×40.

Histopathology revealed a thickened bronchial wall with lymphocyte and macrophage infiltration alongside focal amyloid deposits within the connective tissue and surrounding bronchial glands and cartilage. MM was complicated by focal or polysegmental pneumonia in all patients. Substantial infiltration of lymphocytes, neutrophils and macrophages was observed in the alveolar spaces and interalveolar septa (Figure 2).

**Liver:** Gross examination revealed a firm liver, weighing 900–2500 g, with a gray-brown waxy appearance on section, characteristic of hepatic amyloidosis.

**Figure 3:**
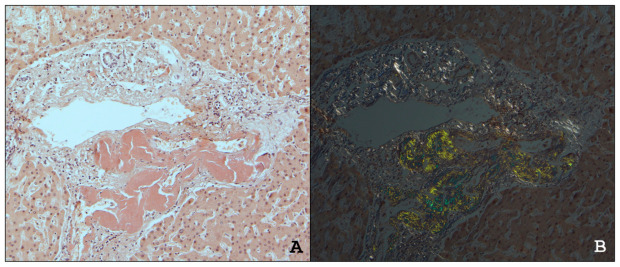
Microscopic image of the liver tissue of a deceased female patient, 68 years of age. (**A**) Amyloid deposits in the hepatic portal tract; (**B**) amyloid masses displaying specific birefringence under polarized light. CR staining, ×100.

Histopathological analysis confirmed extensive Congo-red-positive deposits within the spaces of Disse, between sinusoidal endotheliocytes and hepatocytes (Figure 3). This caused sinusoidal compression, resulting in impaired hepatocyte perfusion and associated metabolic dysfunction.

**Kidneys:** Gross examination demonstrated that the kidneys, weighing 220–320 g, had increased parenchymal firmness, in most cases associated with an indistinct corticomedullary junction. Microscopy revealed diffuse amyloid deposits within the glomerular mesangial matrix and focal glomerular sclerosis. Proteinaceous deposits were also identified along the glomerular basement membrane.

**Figure 4:**
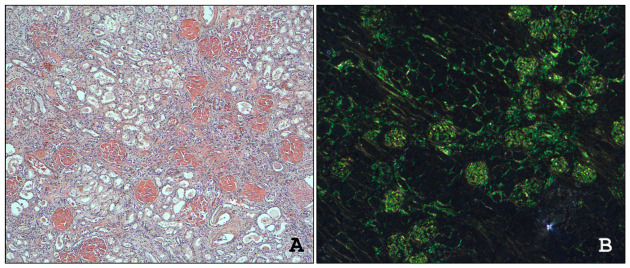
Microscopic image of the renal tissue of a deceased male patient, 71 years of age. (**A**) Amyloid deposits in the glomeruli, vascular walls and stroma. (**B**) Characteristic birefringence under polarized light. CR staining, ×40.

Renal tubules exhibited atrophic changes, contained eosinophilic cylinders in the lumens, and were compressed by amyloid deposition in the GBM, interstitium, and vascular walls (Figure 4). Focal inflammatory round-cell infiltration was observed in the renal stroma. Other findings included dystrophic changes, necrosis, and sclerosis in the tubular epithelium, accompanied by focal calcification (secondary to hypercalcemia).

**Spleen:** Upon gross examination, the spleen was firm, weighing 130–400 g, and displayed a waxy cut surface, characteristic of splenic amyloidosis. Microscopic assessment of the spleen demonstrated atrophic follicles in the white pulp secondary to compression by amyloid deposits in the surrounding tissues. The red pulp volume was expanded and infiltrated with atypical plasma cells. Massive foci of amyloid deposition were noted in the red pulp and between the sinusoids. Congo-red staining confirmed the presence of deposits within the vascular walls.

**Bone marrow:** Macroscopically, the bone marrow in vertebral bodies appeared “juicy” and often reddish-brown. Vertebral bodies, in most cases, were characterized by increased porosity. Furthermore, reddish-gray irregular destructive foci were identified within the vertebral bodies in four autopsies.

**Figure 5:**
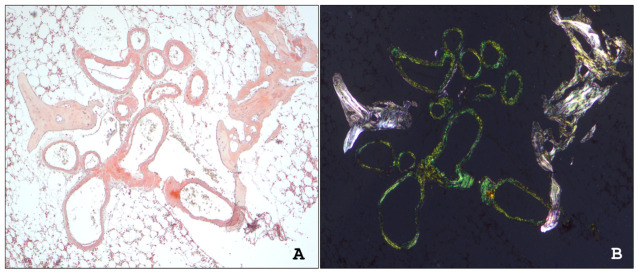
Amyloid deposits in the bone marrow of a patient with MM characterized by monoclonal G-kappa secretion. (**A**) Amyloid deposits in the vascular walls. (**B**) Characteristic birefringence of amyloid deposits in the vascular walls under polarized light. CR staining, ×100.

**Figure 6:**
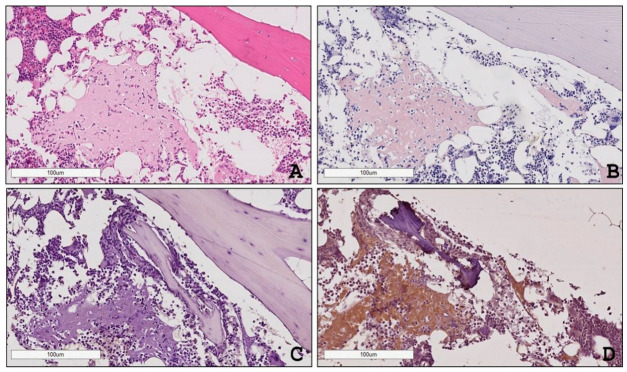
IHC typing of amyloid deposits in the bone marrow of the patient with MM. (**A**) Homogenous eosinophilic amyloid deposits in the interstitium, H&E staining. (**B**) Brick-reddish amyloid deposits, CR staining. (**C**) Negative immunostaining of amyloid deposits with AL-lambda amyloid antibody. (**D**) Positive immunostaining with AL-kappa amyloid antibody ×200.

Microscopic examination revealed clusters of atypical pleomorphic, multinuclear plasma cells displaying varying degrees of maturity, specifically mature plasma cells, plasmablasts and pro-plasmacytes. In most cases, an interstitial–endosteal infiltration pattern of the bone marrow with myeloma cells was observed. An interstitial-nodular pattern of bone marrow infiltration was documented in two autopsies. This pattern was characterized by the coexistence of interstitial infiltrates and nodular myeloma foci in the central regions of the bone marrow spaces. Amyloid deposits were present in the vascular walls and interstitium (Figure 5). IHC typing of amyloid confirmed 18 cases of AL-lambda amyloidosis and six cases of AL-kappa amyloidosis (Figure 6).

**Figure 7:**
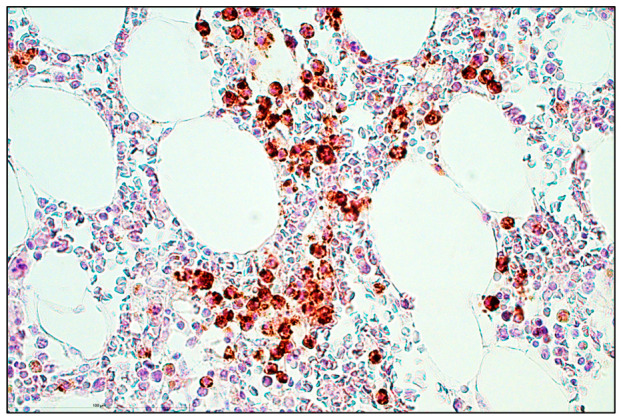
Bone marrow of a patient with MM characterized by monoclonal G-kappa secretion. IHC analysis: CD138 immunoreactivity in myeloma cells, ×200.

IHC staining with CD138 antibody (Syndecan 1) produced a positive reaction in myeloma cells (Figure 7).

## 3. Discussion

The co-occurrence of MM and AL amyloidosis presents a complex clinical and pathological scenario. The reported prevalence varies widely (approximately 8–43%), largely depending on diagnostic criteria and patient selection [10].

Despite differences in their clinical courses, MM and AL amyloidosis are linked by a common etiological substrate: the clonal expansion of the bone marrow plasma cells. In the fifth edition of the *WHO Classification of Haematolymphoid Tumours* (WHO-HAEMS5), both conditions are included in the broad category of plasma cell neoplasms and other diseases with paraproteins. Specifically, immunoglobulin-related (AL) amyloidosis is classified under diseases with monoclonal immunoglobulin deposition, while MM is categorized within plasma cell neoplasms [6,11].

There is a fundamental distinction between MM and AL amyloidosis. MM is characterized by the uncontrolled proliferation of abnormal plasma cells, causing CRAB-related manifestations (hypercalcemia, renal dysfunction, anemia, and destructive bone lesions). Conversely, AL amyloidosis, often occurring with lower bone marrow plasma cell involvement (typically <20% of bone marrow cells), is driven by the qualitative features of secreted monoclonal light chains, which acquire amyloidogenic conformation and deposit in tissues as insoluble fibrils. It appears that the distinction between proliferative and non-proliferative plasma cell clones underpins the divergent histopathological and clinical features of MM and AL amyloidosis.

A central unresolved question in AL-amyloidosis pathogenesis is why only 10–15% of patients with monoclonal free-light-chain (FLC) MM eventually develop AL amyloidosis. The mechanisms preventing amyloidogenic potential from being realized in most cases remain poorly understood and likely involve a complex interplay of interrelated factors:

1. The selection of germline gene as a primary predictor of amyloidogenicity: The choice of light-chain variable domain (IGLV/IGKV) germline gene, encoding the primary amino acid sequence of the variable light (VL) domain, is a key determinant of light-chain amyloidogenicity. Products of certain germline genes exhibit structural instability, predisposing them to misfolding and aggregation. Physiologically, light chains (LCs) are secreted in a stable conformation. However, certain germline genes encode domains with a lower thermodynamic stability, which significantly decreases the energy barrier for their transition to the amyloidogenic state [12,13].

Notably, the λ light-chain isotype is significantly more prevalent in AL amyloidosis than the κ light-chain isotype. The disease is typically characterized by a λ:κ ratio of approximately 3:1, while, in healthy individuals, the normal ratio of κ to λ is closer to 2:1. In AL amyloidosis, the predominance of the λ isotype compared with κ chains is associated with the intrinsically higher amyloidogenic potential of λ light chains. According to the updated AL-Base analysis of 2193 monoclonal light-chain sequences, the genes most enriched in AL amyloidosis comprised the immunoglobulin lambda variable (IGLV) genes, specifically IGLV6-57, IGLV1-36, and IGLV3-1. At the same time, AL-associated κ light chains harbored significantly more mutations than AL-associated λ light chains, which suggests distinct molecular pathways of amyloidogenesis [14]. The high amyloidogenicity of IGLV6-57 lambda chains stems from intrinsic structural features of the variable domain that predispose the protein to misfolding, independently of specific somatic mutations. Thus, the predisposition to fibrillogenesis appears to be a shared characteristic of the entire IGLV6 family rather than a consequence of individual point substitutions.

The amyloidogenic properties of κ chains are driven by the combination of decreased stability of the variable domain and modulating role of the constant domain. Notably, a more substantial reduction in the thermodynamic stability of κ chains is necessary to drive fibrillogenesis compared with that of λ chains. Overall, λ chains exhibit an inherent structural predisposition to fibrillogenesis, whereas κ chains may become amyloidogenic only after accumulating a specific threshold of somatic modifications [15].

It is worth pointing out that, in contrast to AL amyloidosis, patients with MM demonstrate distinct immunoglobulin germline gene repertoires. Certain families, such as immunoglobulin heavy variable 3-30 (IGHV3-30), display lower intrinsic amyloidogenic potential. Thus, the differential gene repertoire between clones causing MM and AL amyloidosis represents a primary molecular distinction in these plasma cell dyscrasias [16,17].

2. Somatic hypermutagenesis—the role of VL-domain destabilizing mutations: Though germline genes dictate a baseline predisposition to amyloidogenicity, the final amyloidogenic potential of a light chain is largely driven by somatic hypermutations accumulated during B-cell affinity maturation. Mutations within the highly variable CDR2 and CDR3 loops can strongly impact the conformational dynamics of the conserved framework regions of the VL domain. This effect arises from deviations from canonical CDR structures induced by even subtle amino acid substitutions. It is suggested that the native CDR loops stabilize the core protein chain through interactions with the framework regions. Mutations that impair these interactions increase fluctuations in the framework regions, facilitating the formation of partially unfolded intermediates that promote the nucleation of amyloid fibrils [18,19]. The fundamental distinction between AL amyloidosis and MM lies in the amyloidogenic potential of the light chains secreted by the plasma cell clone. The monoclonal light chains (LCs) secreted by the myeloma clone, despite somatic mutations, frequently maintain their native conformation without acquiring amyloidogenic properties. Therefore, most patients with MM do not develop AL amyloidosis.
3. Post-translational modifications and additional structural factors: While the pathogenicity of LCs is encoded in their sequence, post-translational modifications (PTMs) represent key additional drivers of their amyloidogenic features. Glycosylation plays an important role in amyloidogenesis: somatic mutations can introduce additional N-glycosylation sites into the kappa-chain variable domain. This impairs interactions with chaperone proteins of the endoplasmic reticulum (ER), induces protein misfolding, and significantly increases aggregation potential.

Glycosylated light chains along with N-terminal pyroglutamylation and altered disulfide bonds destabilize chain conformation, driving predominant amyloid accumulation in the liver and leading to a poorer patient prognosis [20,21,22].

4. Non-fidelity of the plasma cell proteostasis system: Under physiological conditions, thermodynamically unstable LCs are typically recognized, delayed and degraded by the Endoplasmic Reticulum Quality Control (ERQC) pathways, comprising the unfolded protein response (UPR), chaperones (e.g., HSP70, HSP90, GRP78/BiP), and ubiquitin–proteasome system [23]. In AL amyloidosis, a specific proteostasis impairment pattern is observed: dysfunctional UPR factors (ATF6, XBP1) fail to retain misfolded light chains in the ER, resulting in their excessive secretion into the extracellular space [24]. Thus, the severity of proteolytic stress determines the disease nature: the clone, unable to handle the accumulated misfolded chains, releases them into the extracellular space, where fibrillogenesis is initiated [24,25].
5. Amyloidogenic light chain as an intracellular stressor: Another pathogenetic distinction of AL amyloidosis from MM is that the amyloidogenic light chains exert direct proteotoxicity not only on target organs but also on the producer plasma cells. AL amyloidosis presents a paradox where a small, non-proliferating clone produces a highly toxic protein in an amount causing the dysfunction of multiple organ systems in the absence of typical CRAB manifestations [26].

Recent research has found that many AL amyloidosis and MM patients experience significant delays in receiving a clinical diagnosis. The median time from the first consultation to diagnosis was 163 days for MM and 180–441 days for AL amyloidosis [27].

In a study of 1523 patients, Hester et al. found that the median time from symptom onset to AL amyloidosis diagnosis was 2.7 years [28].

Non-specific symptoms and morphological changes that mimic other diseases, such as diabetic nephropathy, hypertensive heart disease, and liver cirrhosis, underpin why AL/MM comorbidity is frequently identified postmortem rather than during the patient’s life [29,30,31]. Postmortem examination is crucial for accurately assessing the prevalence and histopathological characteristics of concomitant MM and AL amyloidosis. However, the existing scientific literature primarily focuses on either condition individually, leaving a significant gap in understanding their combined impact. Thus, systematic data describing the comprehensive spectrum of autopsy findings in organs and tissues is scarce, with the current evidence largely limited to isolated case reports [32,33].

In this study, we analyzed 24 autopsy cases of concomitant MM and AL amyloidosis. In all patients the diagnosis of MM was confirmed during life. However, only in 15 of them (62.5%) was AL amyloidosis revealed prior to death. This type of amyloidosis most commonly co-exists with MM, though other forms of amyloidosis may also co-occur.

According to a retrospective analysis of 1469 bone marrow amyloid biopsies by Chiu et al., AL amyloidosis was identified in 79.8% of cases, while transthyretin amyloidosis accounted for 16.3%; heavy-chain amyloidosis, AA, and amyloid beta-2-microglobulin were confirmed only in rare instances [34].

A recent study demonstrated significant differences in the clinical course of kappa MM with identified bone marrow amyloid deposits (BM-ADs) and lambda MM with BM-ADs. Specifically, lambda MM with BM-AD patients had significantly higher creatinine and albumin levels and a higher incidence of proteinuria, not associated with Bence-Jones protein. Furthermore, the lambda MM with BM-AD group required dialysis more frequently [35].

The development of kappa-type AL amyloidosis secondary to MM is less common, but this comorbidity is characterized by a more severe clinical course, rapidly progressing nephropathy with early onset of chronic renal failure, which exacerbates systemic intoxication and contributes to cardiac decompensation [36].

In addition, AL-associated κ light chains harbor significantly more mutations than those in multiple myeloma. A study by Schreiner et al., including 41 kappa AL and 83 MM patients, demonstrated that IGKV1/D-33-segments of AL had a higher mutation count (AL = 12.0 vs. MM = 10.0), while FR3 and CDR3 were most frequently mutated in both patient groups [37].

These distinctive features underscore significant differences in the pathogenesis and involvement of specific organs in AL and MM, which in the future may influence the choice of treatment paradigms for these patients. The analysis of medical records and autopsy reports in our study demonstrated that renal failure was the most common primary cause of death associated with the amyloid atrophy of the functional tissue.

Focal bronchopneumonia, a common complication of MM, was identified in all our patients. It is suggested that this complication is attributable to the compromised immune status of this patient cohort. One patient developed systemic AL amyloidosis with amyloid cardiomyopathy, resulting in a fatal myocardial infarction and acute cardiac failure.

Accurate interpretation of histopathological changes requires a clear distinction between organ lesions caused by AL amyloidosis and those typical of MM without amyloidosis. For example, in patients with MM without amyloidosis, the most frequent kidney histopathological manifestation was myeloma cast nephropathy (MCN). As demonstrated by Shankar et al. in a retrospective, multicenter study that included 61 patients with MM, the most common histological pattern on kidney biopsy was MCN (72.1%). Morphological characteristics of MCN include the obstruction of renal distal tubules by dense fragmented cylinders formed by light-chain complexes with Tamm–Horsfall glycoprotein, accompanied by a perifocal giant cell reaction. In the same study, AL amyloidosis was confirmed only in 18% of patients and presented with a distinct clinical pattern, primarily characterized by nephrotic syndrome, compared with the acute kidney injury observed in MCN [38].

Cardiac involvement in AL amyloidosis and multiple myeloma (MM) without amyloidosis also requires careful distinction. In MM without amyloidosis, heart lesions are characterized by extramedullary plasmacytoma—myocardial, pericardial, and endocardial infiltration by clusters of atypical plasma cells. These lesions lack amyloid fibrils and produce a negative histochemical reaction upon Congo red staining [39,40]. On the other hand, patients with MM and concurrent AL amyloidosis demonstrate diffuse pericellular amyloid deposits that lead to progressive cardiomyocyte atrophy. These two patterns reveal marked disparities that are apparent even during routine histochemical analysis.

Hepatic involvement in MM is most frequently described by diffuse sinusoidal infiltrates of atypical plasma cells, or as discrete nodules [41]. In contrast, hepatic amyloidosis is characterized by the extracellular amyloid deposition in the spaces of Disse and along the blood vessels, typically occurring without cellular infiltration [42]. These morphological distinctions suggest that our findings are specific to the co-existing MM and AL amyloidosis.

It is worth noting that, in patients with co-existing MM and AL amyloidosis, the prognosis depends not on the presence of MM but mostly on the severity of cardiac amyloidosis. A study by Yu et al. demonstrated that MM-AL patients with cardiac involvement had worse survival than their non-cardiac counterparts, median 5.6 and 41.5 months, respectively [43].

MM-AL patients with a baseline N-terminal pro-brain natriuretic peptide (NT-proBNP) ≥ 8500 pg/mL exhibited a poor median overall survival compared with patients with lower values (6.7 months vs. 79.2 months, respectively) (*p* < 0.001). Univariate analysis identified NT-proBNP ≥ 8500 pg/mL as a significant predictor of poor prognosis (HR 2.09).

A study by Wang et al. demonstrated that concomitant cardiac amyloidosis (MM-AL) significantly worsened prognosis in multiple myeloma patients, with median survival reduced to 15.3 months compared with 41.8 months in those without cardiac involvement [44].

Early diagnosis of cardiac amyloidosis is difficult, as classic manifestations of right-sided congestive heart failure typically emerge only in advanced stages. In our study, cardiac involvement was identified in 19 of 24 autopsy cases. Extensive amyloid infiltration caused cardiomyocyte damage while simultaneously reducing myocardial elasticity and increasing stiffness. The progressive myocardial infiltration impairs physiological contraction, leading to a reduced stroke volume; consequently, while the left ventricle ejection fraction (LVEF) may remain preserved initially, it declines as the infiltration advances [45].

The convergence of pulmonary amyloidosis and multiple myeloma is uncommon. However, in our study amyloid deposits were identified in the pulmonary stroma and vasculature in one-third of autopsy cases. In this connection it is important to stress that pulmonary amyloidosis should be considered a potential cause of hemoptysis in patients with MM [46].

In our study, destructive bone lesions were observed in a quarter of deceased patients. Previous case reports described bone destruction associated with AL amyloidosis in the absence of MM. Amyloid deposits within the bone tissue can trigger protein matrix transformation and subsequent osteoclast activation. Furthermore, amyloid dystrophy of intraosseous vessels impairs tissue nutrition, potentially contributing to progressive bone destruction [47].

In summary, the onset of systemic amyloidosis in patients with MM correlates with a poorer prognosis and reduced therapeutic response. Currently, a significant gap remains in assessing how new therapeutic regimens influence histopathology and organ lesion severity. Also there is a critical need for robust algorithms to evaluate the efficacy of anti-fibril therapy and the prospects of immunomodulatory agents for advancing clinical practice.

Identifying AL amyloidosis at early stages is critical in patients with newly diagnosed MM. Implementing standardized AL screening, incorporating NT-proBNP, renal function, and alkaline phosphatase, may improve early diagnosis of AL amyloidosis across the spectrum of monoclonal gammopathies.

A multidisciplinary approach, involving hematologists, cardiologists, nephrologists, pathologists and general practitioners, is essential for minimizing diagnostic delays and optimizing patient outcomes.

## 4. Materials and Methods

This study includes 24 autopsy cases with confirmed concomitant MM and AL amyloidosis that were selected between 2015 and 2025 from the Amyloid Register of the Scientific Research Institute of Human Morphology named after academician A.P. Avtsyn of the Federal State Budgetary Scientific Institution “Russian Scientific Center of Surgery Named After Academician B.V. Petrovsky.” As a retrospective analysis of archived autopsy records, this study has several limitations. Specifically, clinical data, such as treatment protocols and patient responses to therapy, were unavailable or incomplete. Therefore, our analysis was restricted to the postmortem examination of tissue specimens archived in our amyloid register.

Postmortem examinations were carried out no later than 1.5 days after the pronouncement of death. A complete autopsy was performed in all cases. Fixation in 10% neutral buffered formalin solution and paraffin embedding was used to preserve the obtained tissue samples. Then, a histological examination of the lungs, heart, liver, kidneys, and spleen was carried out. All histological sections were stained with Mayer’s hematoxylin plus eosin (H&E) reagent (BioVitrum company, St. Petersburg, Russia). Congo red stain (CR) was used to detect amyloid deposits (BioVitrum company, St. Petersburg, Russia). To examine the samples, we used a polarized light microscope equipped with a set of fluorescent filters 340–560 nm (Leica DM2500, Leica Microsystems AG, Wetzlar, Germany).

To conduct IHC analysis, tissue sections of 3–5 µm thickness were prepared from paraffin blocks. To perform amyloid typing, the following panel of antibodies was used: anti-serum amyloid P component polyclonal antibody (Anti-Serum Amyloid P/SAP antibody, Abcam, Cambridge, UK); anti-transthyretin polyclonal antibody (Prealbumin, Abcam, Cambridge, UK); anti-AA-amyloid antibody (Clone C3, Cloud-Clone Corp., Katy, TX, USA); anti-AL-kappa monoclonal antibodies (clone CH15, Leica Biosystems, Novocastra™, Newcastle Upon Tyne, UK); and, anti-AL-lambda monoclonal antibodies (clone SHL53, Leica Biosystems, Novocastra™). All bone marrow sections were stained with an anti-human CD138 antibody (clone MI15; Dako, Denmark GlostrupA/S) to identify myeloma (plasma) cells. Immunostaining was performed on formalin-fixed and paraffin-embedded sections with the Bond Max Leica immunostainer, using the Bond Polymer Refine Detection (HRP-DAB) (Leica Microsystems, Wetzlar, Germany). Antigen retrieval was carried out using ER2-Bond Epitope Retrieval Solution 2.

## 5. Conclusions

The clinical severity of MM is driven by this primary condition, and secondary systemic AL amyloidosis is a serious complication. Given that AL amyloidosis often leads to multiorgan failure and increased mortality, screening for its co-occurrence is essential for determining MM treatment strategies.

Our histopathological findings demonstrate that extensive amyloid deposition within the lungs, heart, and kidneys characterizes the morphological basis for multiorgan failure in patients with MM and secondary systemic AL amyloidosis.

Since the clinical course of MM can remain asymptomatic for extended periods, manifestations of organ-related amyloid deposition may predominate. This highlights the necessity of screening patients for the co-occurrence of these conditions.

Early diagnosis of AL amyloidosis in patients with MM is critical for timely therapeutic intervention and improved prognosis. Histopathological analysis remains the primary tool for confirming diagnosis and understanding pathogenetic pathways. These insights are essential for the development of advanced plasma-cell-targeted immunotherapies and novel amyloid-resorbing drugs.

## Data Availability

The original contributions presented in this study are included in the article. Further inquiries can be directed to the corresponding author.

## Abbreviations

The following abbreviations are used in this manuscript:

MM	Multiple myeloma
SMM	Smoldering multiple myeloma
FLCs	Free light chains
IGLV	Immunoglobulin lambda light-chain variable region
IHC	Immunohistochemistry
BM-ADs	Bone marrow amyloid deposits
MGUS	Monoclonal gammopathy of undetermined significance
